# What does “eat more” really mean? - Nutritional changes in food intake during a 6 week ‘Restore’ parent group program.

**DOI:** 10.1186/2050-2974-3-S1-O60

**Published:** 2015-11-23

**Authors:** Jodie Sheraton, Katryna Henry, Judith Leahy

**Affiliations:** 1 Central Coast Eating Disorders Outpatient Service, Wyong, Australia

## 

Refeeding a child back to health from an eating disorder can be difficult for families despite having successfully fed their child prior to the illness. Parents and clinicians push to increase food intake, but the question remains ‘how much is enough for weight gain? This dilemma can result in resistance from the child, conflict, increased stress and lack of confidence for parents.

Our ‘Restore’ ” group parent program evolved from the Westmead Children's Hospital “Nourish” program. It incorporates elements from Multiple Family Therapy developed by Ivan Eisler, Maudsley Family Therapy and is enhanced with nutrition education for parents with concomitant weight and symptom monitoring of the child. The nutrition component is delivered by an Accredited Practising Dietitian and includes: using the RAVES approach, food portions, ‘front loading’ and feeding responsibility.

Two cohorts were evaluated using FoodWorks analysis of pre/post food diaries, behaviour change and weight gain.

Feedback on analysis provided parents insight into the extent eating behaviours affected their childs nutrient intake and weight. This resulted in parents consistently offering often more than double what the child was eating at beginning of treatment.

Parents learnt that they must feed their child ‘like they have never fed them before’.

